# Physical activity promotes the development of cognitive ability in adolescents: the chain mediating role based on self-education expectations and learning behaviors

**DOI:** 10.3389/fpsyg.2024.1383384

**Published:** 2024-11-22

**Authors:** Long Cui, Yumei Xing, Hao Zhou, Jia Qian, Junnan Li, Fei Shen, Yifeng Bu

**Affiliations:** ^1^Institute of Physical Education, Jiangsu Normal University, Xuzhou, China; ^2^Library, Jiangsu Normal University, Xuzhou, China

**Keywords:** physical activity, self-education expectations, learning behaviors, cognitive ability, adolescents

## Abstract

Cognitive ability plays a crucial role in adolescents’ academic performance and subsequent career development. Although previous studies have demonstrated that physical activity, self-education expectations, and learning behaviors positively affect the cognitive development of adolescents, the extent of their influence and their mediating roles require further elucidation. This study is based on tracking survey data from 2,688 adolescents in Chinese households collected in 2018. Multiple linear regression, Propensity Score Matching, and Quantile regression were employed to analyze the impact and heterogeneity of physical activity on adolescents’ cognitive ability. Furthermore, the Bootstrap mediation test was used to explore the mediating roles of self-education expectations and learning behaviors in this process. The results indicate the following: Physical activity significantly promotes adolescents’ cognitive ability; for those with poorer cognitive ability, it exerts a greater impact. Moreover, in addition to its direct effects, physical activity indirectly enhances adolescents’ cognitive ability through the mediation of three factors (self-education expectations, learning behaviors, self-education expectations and learning behaviors). These discoveries offer significant insights into diverse strategies for developing cognitive ability in adolescents, contributing to both theoretical research and practical interventions.

## 1 Introduction

Cognitive ability refers to an individual’s performance in various mental activities, including memory and recall, attention inhibition and focus, information processing speed, and spatial and causal reasoning (Robinson, 2012). Adolescence is a critical period for the rapid development and high plasticity of cognitive ability (Spear, 2013; Fandakova and Hartley, 2020; Laube et al., 2020), and the cognitive development that occurs during this period greatly benefits future academic performance, behavioral habits, career success, and overall life satisfaction (Heckman, 2007; Carson et al., 2016). Therefore, it is important to explore the factors influencing adolescent cognitive development and the strategies to promote it. Research has shown that, in addition to biological factors such as the growth of the nervous system (Tay et al., 2017), adequate nutrition (Datta et al., 2020), and quality sleep (Chen et al., 2021), social-behavioral factors such as physical activity (Erickson et al., 2019), self-efficacy (Demirtaş, 2019), depressive mood (Vieira et al., 2021), and education (Judd et al., 2022) significantly impact adolescent cognitive ability. Among these, physical activity, self-education expectations, and learning behaviors have attracted significant attention in recent years for their roles in the development of adolescent cognitive function. Existing studies have shown that physical activity can significantly enhance adolescent cognitive function by promoting neural growth in brain regions, improving the antioxidant capacity of brain tissue, and increasing brain activation levels (Marmeleira, 2013). Self-education expectations, as an individual’s anticipation of future academic achievements, reflect adolescents’ pursuit of academic success and also play an important role in cognitive development. Studies have demonstrated that adolescents with higher self-education expectations and positive learning behaviors often receive more cognitive training, thereby enhancing cognitive ability (Diamond, 2013). However, there is currently a lack of comprehensive research on the interaction mechanisms between physical activity and self-education expectations in relation to adolescent cognitive ability. Additionally, there are widespread concerns that physical activity might encroach on learning time, triggering debates regarding the relationship between physical activity and learning behaviors. Thus, a comprehensive analysis of the interrelationships between physical activity, self-education expectations, and learning behaviors, along with further exploration of their effects on adolescent cognitive development, is of significant theoretical and practical importance.

Furthermore, the relationships among these factors may differ depending on the cultural context. In China, the educational environment and academic demands placed on adolescents differ significantly from those in other countries, particularly with the high emphasis on academic performance and the high expectations from parents and schools, resulting in greater academic pressure on adolescents and fewer opportunities to engage in physical activity. Therefore, exploring the relationships between physical activity, self-education expectations, learning behaviors, and cognitive ability development among adolescents in the Chinese cultural context not only enriches research across diverse cultural backgrounds but also provides scientific evidence for formulating physical activity policies tailored to the realities of Chinese adolescents. It is noteworthy that existing studies often focus on specific age groups or grade levels (Cancela et al., 2019; Fang and Huang, 2021; Shigeta et al., 2021), with few comprehensive studies treating students from different grade levels as a whole. Given that adolescence is a critical period for cognitive ability development and behavioral shaping, conducting systematic analysis by treating upper elementary, middle, and high school students as a continuum of development provides a more comprehensive understanding of the continuity and systematic nature of adolescent cognitive ability development, thereby offering a broader perspective for examining the interplay between physical activity, self-education expectations, and learning behaviors.

Therefore, this study aims to clarify how physical activity, in the Chinese context, promotes the development of adolescents’ cognitive ability by enhancing self-education expectations and improving learning behaviors, thereby contributing to the body of research on the impact of physical activity on adolescent cognitive ability.

### 1.1 Physical activity and cognitive ability

Numerous studies have shown a significant positive correlation between physical activity and the improvement of adolescent cognitive ability. Particularly in Western cultural contexts, multiple cross-sectional studies have demonstrated that higher levels of physical activity are closely related to superior cognitive function and executive abilities (Engeroff et al., 2018; Reigal et al., 2020). Longitudinal studies have also demonstrated similar findings, with one meta-analysis revealing that individuals who engage in higher levels of physical activity experienced a 38% slower rate of cognitive decline over a 12-year follow-up period compared to those who did not participate in physical activity (Sofi et al., 2011). These studies clearly illustrate the long-term protective effect of physical activity on cognitive function. Although research on physical activity and adolescent cognitive development in Western contexts is abundant, related studies based on the Chinese cultural context remain relatively limited and are primarily focused on randomized controlled trials. For example, Chen et al. (2016) showed that a 3-month physical activity intervention significantly improved the executive function of 25 obese adolescents. Similarly, Fu and Fan (2016) conducted an 8-week exercise intervention on 250 adolescents and found that moderate-intensity physical activity significantly improved inhibitory function and shifting ability. These studies suggest that, even in different cultural contexts, physical activity has a significant positive impact on adolescent cognitive ability.

Furthermore, different types of exercise may have varying specific effects on cognitive function. For instance, aerobic exercise is especially effective at improving performance on complex cognitive tasks such as working memory, executive function, and response control, especially in N-back tasks and Stroop tests, where it significantly improves both working memory and information processing speed (Hill et al., 2019). At the same time, resistance training has also shown positive effects on tasks involving selective attention and inhibitory control (Alves et al., 2012). Thus, while different types of physical activity may affect specific components of cognitive function differently, overall, physical activity has a positive effect on cognitive function.

### 1.2 The mediating role of self-educational expectations

Generally, adolescents engaging in higher levels of physical activity exhibit elevated self-confidence and self-efficacy (Laube et al., 2020), possess increased self-educational expectations (Fredricks and Eccles, 2006), and are more likely to attain greater academic achievement (Donnelly and Lambourne, 2011). Studies indicate that the development of motor skills and athletic accomplishments during physical activity can enhance adolescents’ self-worth and self-esteem (Gilani and Dashipour, 2016), which, when transferred to the academic realm, manifest as heightened self-educational expectations. These self-educational expectations further enhance adolescents’ efficiency in cognitive exercises and learning processes through motivational activation, ultimately leading to superior cognitive ability in those with higher expectations (Liu et al., 2022). Consequently, therefore, this study hypothesized that self-educational expectations mediate the relationship between physical activity and cognitive ability.

### 1.3 The mediating role of learning behaviors

Research indicates that cognitive exercise is an effective way to enhance adolescents’ cognitive ability, and suggests that the act of learning itself is the most effective method of cognitive exercise. Many parents hold the belief that physical activity not only diminishes adolescents’ study time, but also adversely affects their academic engagement (Ferreira Silva et al., 2022), Consequently, they often discourage or even prohibit adolescents from participating in physical activity. However, studies have found that individuals engaging in regular physical activity exhibit a stronger sense of purpose and higher levels of self-discipline. This trait contributes to behavioral habits, thereby promoting more learning behaviors in adolescents (Wilson et al., 2019). This in turn enhances cognitive ability and academic performance (Diamond, 2013). Therefore, this study hypothesized that learning behaviors mediate the relationship between physical activity and cognitive ability.

### 1.4 Chain-mediated effects of self-educational expectations and learning behaviors

Expectations significantly influence behavior, and an individual’s thinking can have a direct impact on the decision-making process and motivation, all of which can affect an individual’s behavioral choices (Beckmann and Heckhausen, 2018). Individuals are influenced by subjective thoughts when deciding to engage in a specific behavior, and when the pressure from expectations is perceived, they tend to invest more time in seeking to meet these expectations (Teixeira et al., 2012). Higher self-educational expectations imply encouragement, protection, and recognition of learning-related behaviors, thereby strengthening individuals’ engagement in learning (Bates and Anderson, 2014). Consequently, adolescents’ self-educational expectations can influence behavioral choices and encourage increased learning behaviors. Physical activity, self-educational expectations, and learning behaviors all influence cognitive ability, and physical activity, in turn, is associated with self-educational expectations and learning engagement. Therefore, this study further hypothesized that Self-educational expectations and learning behaviors exert a chain-mediated effect in physical activity influencing adolescents’ cognitive ability.

### 1.5 The current study

**Figure 1:**
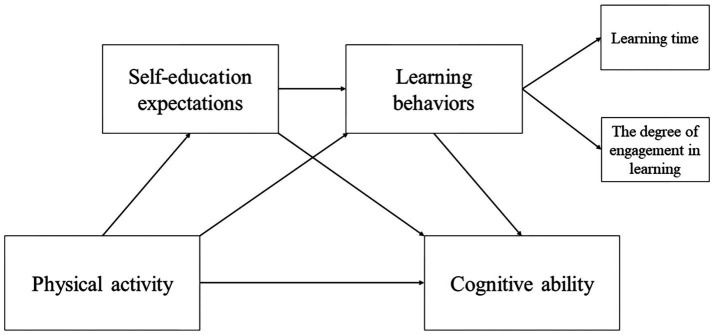
The proposed chain-mediation model.

Adolescents’ cognitive ability significantly contributes to their academic performance and subsequent career development. Thus, improving adolescents’ cognitive ability through approaches grounded in behavioral science and psychology is of paramount importance. Based on an analysis of the relationship between physical activity, self-education expectations, learning behaviors, and adolescent cognitive ability, a chain mediation model was constructed (Figure 1), and four hypotheses were proposed:

*Hypothesis 1*: Physical activity significantly influences the cognitive ability of adolescents.

*Hypothesis 2*: Self-education expectations mediate the relationship between physical activity and cognitive ability.

*Hypothesis 3*: Learning behaviors play a mediating role between physical activity and cognitive ability.

*Hypothesis 4*: Self-education expectations and learning behaviors exert a chained mediating effect in the process whereby physical activity influences cognitive ability in adolescents.

## 2 Materials and methods

### 2.1 Data source and study population

Data was obtained from the China Family Panel Studies (CFPS)^1^, a significant survey program conducted by the Institute of Social Sciences at Peking University. The CFPS sample included participants from 25 provinces, municipalities, and autonomous regions in China, representing approximately 95% of the nation’s total population. Consequently, the CFPS is considered a nationally representative sample (Xie and Lu, 2015), and the survey received approval from the Biomedical Ethics Committee of Peking University (Approval No.: IRB00001052-14010).

The 2018 China Family Panel Studies (CFPS) data was utilized for this study, as the onset of the COVID-19 pandemic in 2020 led to a reduction in face-to-face interviews, resulting in significant missing cognitive data from that year’s CFPS. The database encompassed 37,354 participants, comprising 3,690 adolescents from upper elementary, middle, and high schools. Of these, 691 participants lacked cognitive data and 311 were absent of other study variables. The final sample size that met the study’s requirements consisted of 2,688 adolescents aged between 9 and 20.

### 2.2 Variables

#### 2.2.1 Cognitive ability

The cognitive ability assessment in CFPS 2018 employed vocabulary and math tests designed by the CFPS research team. The CFPS cognitive ability tests have shown high reliability and validity (Qiong and Peihua, 2016) and have been widely validated and applied in previous studies to measure cognitive ability in Chinese adolescents and adults (Zhang et al., 2018; Ren et al., 2019; Li et al., 2021). The CFPS cognitive ability test scale was designed based on the Guttman scale in psychometrics, with testing procedures similar to corresponding subtests of the Wechsler Intelligence Scale, with all vocabulary and math test items drawn from standard Chinese primary and secondary school textbooks (Huang et al., 2015). Specifically, the vocabulary test required respondents to read aloud from one of eight randomly selected vocabulary lists, with each list containing 34 words. The test concluded when the respondent failed to read or misread three consecutive words, or when they completed the 34th word. The final score for the vocabulary test ranged from 0 to 34. In the math test, respondents randomly selected one of four sets of math problems, which covered topics such as addition, subtraction, multiplication, division, trigonometric functions, and permutations and combinations. The test ended when the respondent failed to answer or answered incorrectly three consecutive questions, or after completing all 24 problems, with the final score ranging from 0 to 24. The total cognitive ability score was the sum of the vocabulary and math test scores, with a range from 0 to 58 (Shi et al., 2022).

#### 2.2.2 Physical activity

Physical activity assessment was based on the average time an individual spent exercising each day. The CFPS survey queried young respondents about their physical activity participation, measuring it through two time-based questions: “Number of times exercised in the past week” and “Total hours exercised in the past week.” In accordance with related literature (Zhang, 2023), this study initially excluded outliers, including participants who reported “more than 360 min of exercise per session” and those who had not graduated from elementary school by age 15, middle school by age 18, and high school by age 21. Subsequently, the study calculated the average daily exercise duration in minutes using the formula: Average daily exercise time (minutes) = [Weekly exercise time (minutes)/7], to determine the average daily exercise time for adolescents. In order to make the explanatory variables more consistent with the requirements of normal distribution and to ensure that samples with 0 daily exercise time were not excluded, the study initially incremented the daily exercise time (in minutes) by 1, and subsequently transformed it into a natural logarithm, resulting in the continuous variable that represents adolescents’ Physical activity duration (Hu and Yu, 2019; Zhong et al., 2022).

#### 2.2.3 Self-education expectations

In the CFPS survey, respondents were queried about the minimum level of education they believed they should complete. Responses fell into eight categories: “no need to study,” “elementary school,” “junior high school,” “high school,” “college,” “undergraduate,” “master’s degree,” and “doctoral degree,” each response was received a score ranging from 1 to 8, with higher scores indicating greater self-educational expectations.

#### 2.2.4 Learning behaviors

This study, based on existing literature and CFPS data, measures learning behaviors along two dimensions: the first is the “quantity” of learning behaviors, specifically the time spent on learning; the second is the “quality” of learning behaviors, which refers to the degree of engagement in learning (Chen et al., 2015; Wang et al., 2024). Learning time was assessed based on average daily learning time (minutes), and the CFPS survey queried respondents regarding their learning time, primarily measured through two time-dimension questions: “non-weekend learning time” and “weekend learning time.” In this paper, the formula employed to calculate the average daily learning time of adolescents is expressed as Average daily learning time (minutes) = ((non-weekend learning time * 5 + weekend learning time * 2)/7). When using this variable, each daily learning time value was first incremented by 1 and then log-transformed to smooth the data. The degree of engagement in learning was evaluated using a self-rating scale consisting of 5 questions, specifically: “I study hard,” “I concentrate on my studies in class,” “I follow school rules and discipline,” “I like to keep my belongings neatly organized at school,” and “I only play after I have completed my schoolwork,” with ratings ranging from 1 (strongly disagree) to 5 (strongly agree). A higher score indicates a stronger level of agreement. The Cronbach’s alpha coefficient for this scale in 2018 was 0.70, indicating acceptable internal consistency for the Learning Engagement Self-Rating Scale. Owing to the large number of missing data for the degree of engagement in learning variable, this paper uses data from the previous period (2016 CFPS survey) to impute missing values, and the sample size of those who answered the degree of engagement in learning question after imputation was 1965.

#### 2.2.5 Control variables

Based on previous studies, this study selects various control variables, encompassing respondents’ individual characteristics, family characteristics, and school characteristics. Individual characteristic variables include age, gender (1 for Male, 0 for Female), Residence (0 for rural, 1 for urban), and educational level (1 for elementary school, 2 for junior school, and 3 for senior school), household characteristic variables include parental education level (educational attainment level), household expenditures (expressed as the logarithm). School characteristics cover the school location, whether it is a public school, whether it is a key school and class size. Results for variable naming and descriptive statistics are presented in Table 1. In the regression analysis, all categorical variables are represented as dummy variables.

**Table 1:** The definition of variables and descriptive statistical results.

Variable name	Variables definition	Mean	SD	*N*
Cognitive ability	Cognitive ability test scores	38.22	10.39	2,688
Physical activity	Logarithm of average daily physical activity time	2.30	1.79	2,688
Self-education expectations	Level of education	5.36	1.23	2,688
Learning time	Logarithm of average daily learning time	6.03	0.42	2,688
The degree of engagement in learning	The degree of Engagement in Learning Scale Score	22.30	2.99	1965
Age	Age	13.73	2.66	2,688
Gender	Male = 1, Female = 0	0.53	0.50	2,688
Residence	Urban = 1, Rural = 0	0.42	0.49	2,688
Education level	1 = Elementary school, 2 = Junior school, 3 = Senior school	1.79	0.79	2,688
Parental education level	Level of education	3.01	1.18	2,688
Household expenditures	Logarithm of annual household expenditures	10.92	0.79	2,688
School location	1 = Provincial capital city, 2 = General city, 3 = County town, 4 = Countryside	3.21	0.92	2,688
Public School Status	1 = Public school, 0 = Private school	0.91	0.28	2,688
Key School Status	1 = key school, 0 = non-key school	0.25	0.43	2,688
Class size	Number of people in the class	48.13	15.75	2,688
Physical activity status	1 = regular physical activity, 0 = Infrequent physical activity	0.50	0.50	2,688

### 2.3 Methodology specification

(1) The Ordinary Least Squares (OLS) method was initially employed to estimate the impact of physical activity on cognitive ability. The model was constructed using the following formula:


(1) $$CA_{i}=a+\beta_{1}\mathrm{Exercis}e_{i}+\beta_{2}\mathrm{Contro}l_{i}+\in_{i}$$

In Equation (1), CA_i_ represents the cognitive ability score of Individual i, Exercise_i_ represents Individual i’s physical activity level, and Control_i_ denotes the control variable. The term *a* is the intercept, *β*_1_ is the coefficient for the physical activity variable Exercise, *β*_2_ is the coefficient for the control variable, and *ε*_i_ represents the error term.

(2) In sociological research, data often come from observations and surveys. Because many other variables confound the relationship between the independent and dependent variables, interventions are not randomly assigned to study subjects, which can lead to sample selection bias in OLS regression. Specifically in this study, whether individuals engage in physical activity is not randomly assigned but is influenced by a series of covariates (such as gender, age, socioeconomic status), which not only affect the probability of participating in physical activity but may also influence cognitive ability. Even if these covariates are controlled for in OLS regression, estimation bias may still occur due to an imbalance in covariate distribution, affecting the accuracy of estimating the impact of physical activity on cognitive ability. Propensity Score Matching (PSM) can effectively address this issue. The core principle of propensity score matching is to match individuals who received treatment with those who did not, matching individuals with similar propensity scores in the control group. Since potential confounding variables are controlled during matching, the matched treatment and control groups no longer exhibit significant differences in characteristics, thereby reducing selection bias. This process ensures covariate balance between the treatment and control groups, reducing confounding bias due to covariate differences (Haukoos and Lewis, 2015). Therefore, in this study, we use PSM to test the robustness of the OLS model. The model formula is as follows:


(2) $$ATT=E\left\{E\left[CA_{1i}|,D_{i}=1|,p\left(x_{i}\right)\right]-E\left[CA_{0i}|,D_{i}=0|,p\left(x_{i}\right)\right]\right\}$$

In Equation (2), CA_1i_ represents the cognitive ability score of adolescents who regularly engage in physical activity, while CA_0i_ represents the cognitive ability score of adolescents who do not regularly engage in physical activity. When individual i regularly participates in physical activity, *D*_i_ = 1, otherwise *D*_i_ = 0. *p*(*X*_i_) represents the probability of individual *i* engaging in physical activity. In conducting propensity score matching, the Logit model is first used to calculate the conditional probability, or propensity score, of the treatment group based on the control variables selected in this study. Then, using the estimated propensity scores, individuals in the treatment group who regularly participate in physical activity are matched with individuals in the control group who lack physical activity, ensuring similarity in covariates between the two groups and reducing selection bias. This study employs three commonly used matching methods—nearest neighbor matching, nearest-neighbor matching with caliper, and kernel matching—to estimate the average treatment effect (ATT) of physical activity on cognitive ability. After matching, we evaluate the quality of the matching using two methods: comparing the two-sample *t*-tests of individual covariates and comparing the changes in the kernel density function of propensity scores before and after matching.

(3) Quantile regression was employed to investigate if the impact of physical activity on cognitive ability varies across different cognitive ability distributions. Quantile regression is effective in delineating the impact of explanatory variables on dependent variables at varying quantiles. The model is formulated as follows:


(3) $$Q\mathrm{uantil}e_{\tau }\left(CA\right)=a_{\tau }+\beta_{1\tau }\mathrm{Exercis}e_{i}+\beta_{2\tau }\mathrm{Contro}l_{i}+\in_{i\tau }$$

In Equation (3), *a*_T,_ *β*_1T,_ *β*_2T_, and *ε*_iT_ denote the parameters at the T quantile, respectively.

(4) This study uses the bias-corrected percentile Bootstrap method proposed by Hayes (2017) to test the mediation effect, with 5,000 bootstrap samples. In practice, the Process plugin in SPSS is employed, with model 6 selected, while controlling for covariates at the individual, family, and school levels. Physical activity is used as the independent variable, cognitive ability as the dependent variable, and self-education expectations and learning behaviors as mediating variables to establish a chain mediation model. Hypotheses 2–4 of this study are tested, and when the 95% CI of the test results does not include zero, the mediation effect is considered significant.

## 3 Results

### 3.1 The relationship between physical activity and adolescents’ cognitive ability

Table 2 presents the OLS regression results. Model (1) includes only the core variables: physical activity and individual characteristics. The parameter estimate of β1 in Model (1) is estimated at 0.354 (*p* < 0.01), suggesting that physical activity significantly influences cognitive ability. Models (2) and (3) extend the analysis by including family and school characteristics, with the parameter estimate of β1 at 0.273 (*p* < 0.01), suggesting a 0.273-point increase in cognitive ability for every 1% increase in physical activity time. Furthermore, the *R*^2^ value of the regression increased from 0.468 to 0.496, demonstrating an enhanced ability of the model to explain variations in adolescents’ cognitive ability due to the inclusion of family and School characteristics. Consequently, Hypothesis H1 is confirmed.

**Table 2:** The relationship between physical activity and adolescents’ cognitive ability: results of OLS.

Variables	Model (1)	Model (2)	Model (3)
Physical activity	0.354*** (0.083)	0.268*** (0.081)	0.273*** (0.081)
Individual characteristics	Control	Control	Control
Family characteristics		Control	Control
School characteristics			Control
Constant	9.149*** (1.139)	3.298 (2.323)	4.365* (2.645)
*R*^2^	0.468	0.491	0.496

*N* = 2,688, ***p < 0.01, **p < 0.05, *p < 0.1. Standard errors are in parentheses.

### 3.2 Heterogeneity in the effects of physical activity on adolescents’ cognitive ability

#### 3.2.1 Robustness tests for the role of physical activity: estimation based on the PSM

To estimate the net effect of physical activity on cognitive ability, this study employed the propensity score matching (PSM) method to mitigate potential sample selectivity bias that could undermine the reliability of the results. This study defined adolescents participating in physical activity three or more times per week as the “regular physical activity” treatment group, and those with less than three weekly sessions as the “physically inactive” control group (Fang and Huang, 2021). Physical activity was converted into a 0, 1 dummy variable (1 for the treatment group, 0 for the control group), thus satisfying the requirements of the Propensity Score Matching method for the variable.

**Figure 2:**
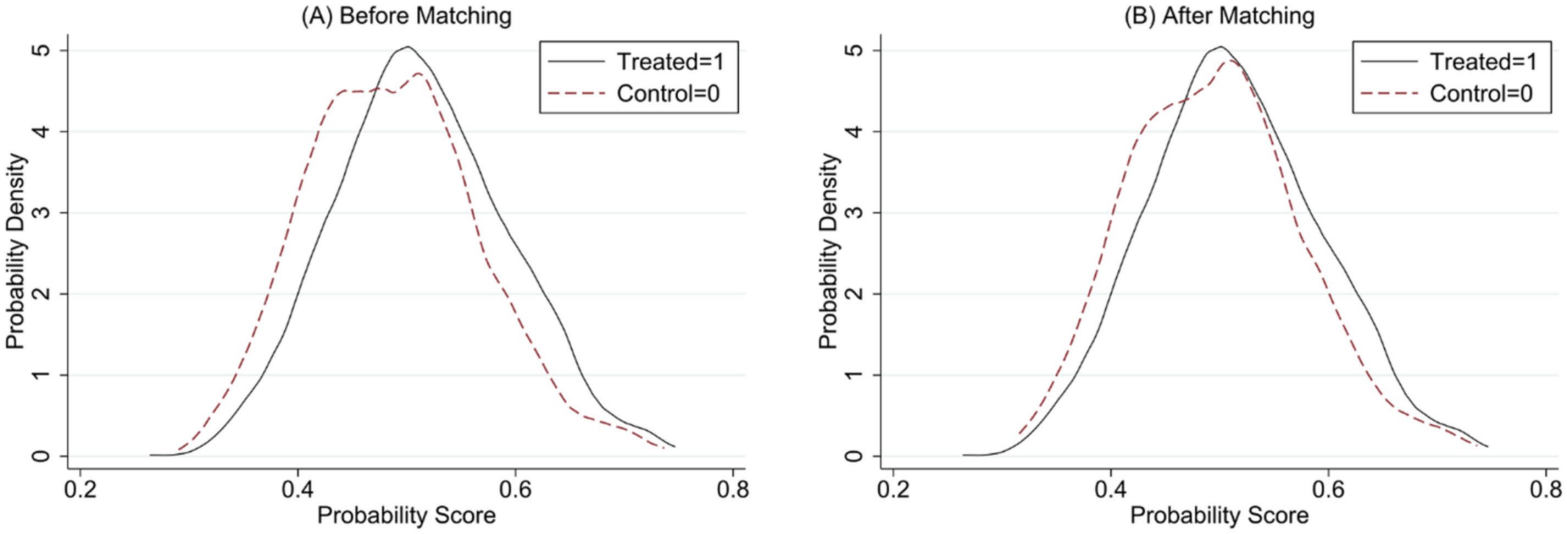
Kernel function plot before and after sample matching. The solid line represents the treatment group, and the dashed line represents the control group.

The study used two methods to test for match quality. First, two-sample t-tests for individual covariates were conducted. The results showed that after propensity score matching, the standardized deviations of all variables were less than 10% and there was no significant difference after matching (*p* > 0.1). This shows that the propensity score matching method solves the sample selectivity bias problem to a large extent. Then compare the changes in the kernel density function distribution of the propensity score values before and after matching. Figure 2 shows the kernel density function plots before and after sample matching. From the figure, it can be observed that the difference in the probability distribution of the propensity score values of the treatment group and the control group after matching is narrowed, the common range of values is wider, and the trend of the curves tends to be more consistent. It indicates that the matching effect is well.

In order to accurately estimate the Average Treatment Effect of Treated on adolescents’ cognitive ability by regular participation in physical activity, three matching methods were used in this study for the test: nearest-neighbor matching (*k* = 2), nearest-neighbor matching with caliper (*k* = 2; *r* = 0.01), and kernel matching (default kernel function and bandwidth). The results showed that regular participation in physical activity consistently contributes significantly to the cognitive ability of adolescents, with effect values ranging from 0.802 to 1.117 (Table 3). The results of all three matching methods are significant at the 5% statistical level, which indicates the robustness of the results obtained through propensity score matching.

**Table 3:** The relationship between physical activity and adolescents’ cognitive ability: results of PSM estimation.

Matching method	T	C	ATT	Standard errors
Nearest-neighbor matching	38.737	37.620	1.117**	0.477
Nearest-neighbor matching with caliper	38.737	37.620	1.117**	0.477
Kernel matching	38.741	37.939	0.802**	0.410

*N* = 2,688, ***p < 0.01, **p < 0.05, *p < 0.1, nearest-neighbor matching (*k* = 2), nearest-neighbor matching with caliper (*k* = 2; *r* = 0.01), kernel matching (default kernel function and bandwidth).

#### 3.2.2 The relationship between physical activity and cognitive ability in adolescents: the influence of individual cognitive level

The Ordinary Least Squares (OLS) analysis quantifies the average effect of physical activity on cognitive ability. To delve into potential differences in the impact of physical activity on students with varying cognitive ability and their distinct manifestations, quantile regression was utilized to examine the heterogeneity in the effects of physical activity.

**Figure 3:**
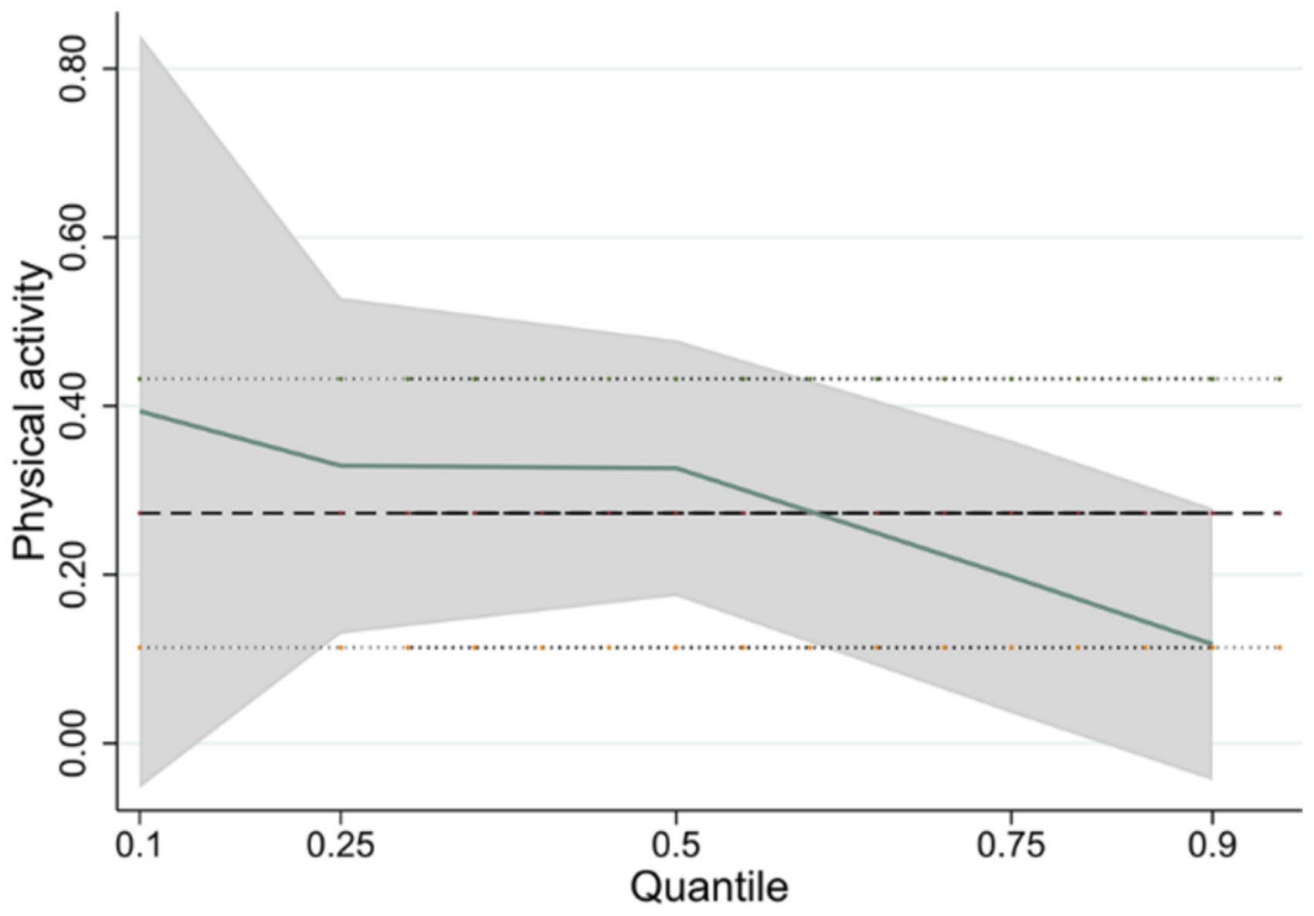
Changes in the physical activity at different percentiles of cognitive ability.

The estimates indicate that the impact of physical activity on cognitive ability, across the 0.1, 0.25, 0.5, 0.75, and 0.9 quartiles, are 0.394 (*p* < 0.1), 0.329 (*p* < 0.01), 0.326 (*p* < 0.01), 0.198 (*p* < 0.01), and 0.118 (*p* < 0.1), respectively (Table 4). According to the estimates across different cognitive ability quartiles, the impact of physical activity on cognitive ability is more pronounced for individuals in the lower cognitive quartiles than those in the higher quartiles (Figure 3), suggesting that adolescents with lower cognitive ability benefit more substantially from physical activity, and the duration of such activity is positively correlated with the extent of cognitive enhancement. Therefore, it is significant to encourage and guide adolescents with lower cognitive ability to actively engage in physical activity, as it promotes cognitive development within this demographic.

**Table 4:** Quantile regression of physical activity on adolescents’ cognitive ability.

Variables	*τ* = 0.1	*τ* = 0.25	*τ* = 0.5	*τ* = 0.75	*τ* = 0.9
Physical activity	0.394* (0.235)	0.329*** (0.125)	0.326*** (0.085)	0.198*** (0.073)	0.118* (0.060)
Individual characteristics	Control	Control	Control	Control	Control
Family characteristics	Control	Control	Control	Control	Control
School characteristics	Control	Control	Control	Control	Control
Constant	−12.476* (6.484)	−6.274 (4.239)	2.854 (2.447)	10.722*** (2.241)	19.852*** (2.853)
*R*^2^	0.245	0.294	0.354	0.375	0.404

*N* = 2,688, ***p < 0.01, **p < 0.05, *p < 0.1. Standard errors are in parentheses.

### 3.3 Chain mediation analysis of self-educational expectations and learning behaviors

Given that this study categorizes learning behaviors into two dimensions: “quantity” referring to learning time, and “quality” indicating the degree of engagement in learning, the mediation analysis should accordingly be conducted in two distinct segments: learning time and the degree of engagement in learning.

#### 3.3.1 Chain mediation analysis of self-education expectations and learning time

**Figure 4:**
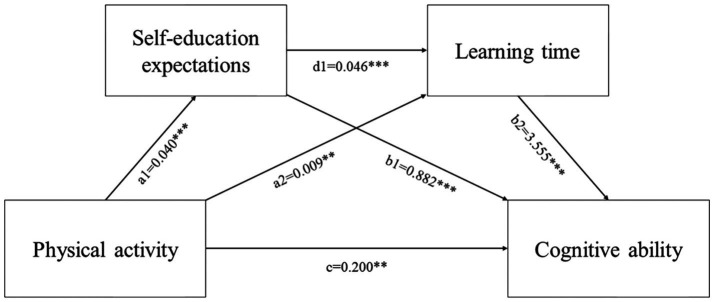
The mediation model of self-educational expectations and learning time as mediators between physical activity and cognitive ability. ***p < 0.01, ***p < 0.05, *p < 0.1, the mediating effect of self-educational expectations on the relationship between physical activity and cognition is denoted as a1*b1; the mediating effect of learning time, as a2*b2; the combined mediating effect of educational expectations and learning time, as a1*d1*b2. The total effect of physical activity on cognition is represented by a1*b1 + a2*b2 + a1*d1*b2 + c.

The results reveal that physical activity exerts a direct effect on cognitive ability, with an estimated impact of 0.1999 (CI: 0.0453–0.3545), after controlling for covariates, contributing to 73.223% of the total effect. The estimated parameter for the indirect effect of physical activity on cognitive ability, mediated by self-educational expectations and learning time, is 0.0730 (CI: 0.0332–0.1205), representing 26.740% of the total effect. The mediation analysis reveals that self-education expectation (physical activity path → self-education expectation path → cognitive ability path) constitutes 12.857% (CI: 0.0119–0.0615) of the total effect, signifying a significant mediation in the relationship between physical activity and cognitive ability, thereby confirming Hypothesis H2. Learning time’s mediating effect (physical activity pathway → learning time pathway → cognitive ability pathway) was found to be 11.538% (CI: 0.0028–0.0673), underscoring its significant role in mediating the relationship between physical activity and cognitive ability. The combined mediation of self-education expectation and learning time (physical activity path → self-education expectation path → learning time path → cognitive ability path) contributed to 2.381% (CI: 0.0021–0.0121) of the effect, indicating a chained mediating role for self-educational expectations and learning time. Table 5 and Figure 4 display the mediation analysis results.

**Table 5:** Mediating role of self-educational expectations and learning time in the relationship between physical activity and cognitive ability.

Effect	Estimate	BootSE	BootLLCI	BootULCI	Effect value (%)
Total effect (physical activity)	0.2730	0.0812	0.1136	0.4323	
Direct effect (physical activity)	0.1999	0.0788	0.0453	0.3545	73.223%
Indirect effect (self-education expectation)	0.0351	0.0127	0. 0119	0.0615	12.857%
Indirect effect (learning time)	0.0315	0.0162	0.0028	0.0673	11.538%
Indirect effect (self-education expectation and learning time)	0.0065	0.0026	0.0021	0.0121	2.381%
Total indirect effect of mediators	0.0730	0.0221	0.0332	0.1205	26.740%

*N = 2,688, BootSE represents the standard error, BootLLCI and BootULCI denote the lower and upper bounds, respectively, of the indirect effect within the 95% confidence interval, as estimated by the bias-corrected percentile Bootstrap method.*

#### 3.3.2 Chain mediation of self-educational expectations and the degree of engagement in learning

**Figure 5:**
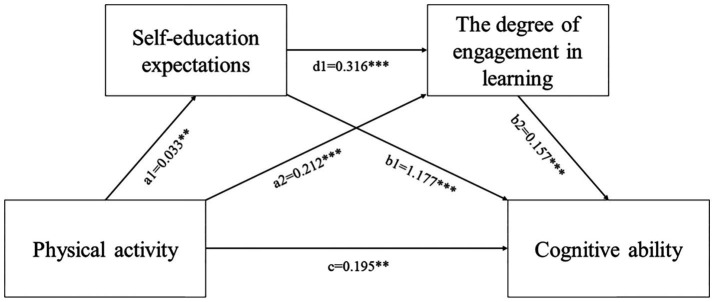
The mediation model of self-educational expectations and the degree of engagement in learning as mediators between physical activity and cognitive ability. ***p < 0.01, ***p < 0.05, *p < 0.1, the mediating effect of self-educational expectations on the relationship between physical activity and cognition is denoted as a1*b1; the mediating effect of the degree of engagement in learning, as a2*b2; the combined mediating effect of educational expectations and the degree of engagement in learning, as a1*d1*b2. The total effect of physical activity on cognition is represented by a1*b1 + a2*b2 + a1*d1*b2 + c.

The mediation effect of the degree of engagement in learning (physical activity path → the degree of engagement in learning path → cognitive ability path) constitutes 12.370% of the overall mediation effect (CI: 0.0083–0.0644), indicating significant mediation by the degree of engagement in learning between physical activity and cognitive ability (Table 6 and Figure 5). Given that the mediation effects of both learning time and the degree of engagement in learning are validated, Hypothesis H3 is confirmed. The combined mediation effect of self-education expectation and learning time (physical activity path → self-education expectations path → the degree of engagement in learning path → cognitive ability path) was 0.594% (CI: 0.0001–0.0044), suggesting that self-educational expectations and the degree of engagement in learning exert chained mediating effects. Given the chain mediating effects of self-educational expectations with both learning time and the degree of engagement in learning are valid, Hypothesis H4 is substantiated.

**Table 6:** Mediating role of self-educational expectations and the degree of engagement in learning in the relationship between physical activity and cognitive ability.

Effect	Estimate	BootSE	BootLLCI	BootULCI	Effect value (%)
Total effect (physical activity)	0.2692	0.0918	0.0892	0.4492	
Direct effect (physical activity)	0.1954	0.0908	0 0.0174	0.3734	72.585%
Indirect effect (self-education expectation)	0.0388	0.0180	0. 0045	0.0774	14.413%
Indirect effect (the degree of engagement in learning)	0.0333	0.0145	0.0083	0.0644	12.370%
Indirect effect (self-education expectation and the degree of engagement in learning)	0.0016	0.0011	0.0001	0.0044	0.594%
Total indirect effect of mediators	0.0738	0.0241	0.0278	0.1228	27.415%

*N = 2,688, BootSE represents the standard error, BootLLCI and BootULCI denote the lower and upper bounds, respectively, of the indirect effect within the 95% confidence interval, as estimated by the bias-corrected percentile Bootstrap method.*

Given the partial missing data for the degree of engagement in learning variable (*n* = 1965), slight discrepancies exist between the data in Table 6 and Figure 5, and those in Table 5 and Figure 4, yet these minor discrepancies also suggest that the missing data for the degree of engagement variable occurs completely at random, not compromising the overall data credibility.

## 4 Discussion

### 4.1 Effects of physical activity on cognitive ability

The findings of the study demonstrate that, after controlling for the variables of individual characteristics, family characteristics and school characteristics, physical activity exerts a significant impact on adolescents’ cognitive ability, and that every 1% increment in physical activity time leads to a 0.273-point enhancement in cognitive ability, corresponding to 0.714% of the average cognitive ability. Considering the issue of sample selection bias, this study employs the Propensity Score Matching method to address potential endogeneity, with the results indicating that the positive impact of physical activity on adolescents’ cognitive ability is robust. This aligns with the conclusions of prior research (Guan and Tena, 2022). An additional significant discovery is the heterogeneity in the impact of physical activity on cognitive ability, as revealed by Quantile regression analysis. This analysis demonstrates that adolescents with lower cognitive ability experience greater benefits from engaging in physical activity, indicating that those at a cognitive disadvantage gain more from physical activity. This diverges from prior findings, which identified the most substantial facilitating effects of physical activity on adolescents with median cognitive ability (Fang and Huang, 2021), and this discrepancy could be due to the younger average age of the samples used in this study. Adolescents, owing to variations in neurological development rates, are more likely to exhibit gaps in cognitive ability, which physical activity can help mitigate. This study illustrates that physical activity more significantly benefits adolescents with lower cognitive ability, highlighting the importance of physical activity in bridging developmental cognitive gaps.

In practice, adolescents with lower cognitive ability are disadvantaged along with those with lower academic performance. If an activity is particularly beneficial to the group possessing disadvantaged cognitive ability, this indicates that such an activity fosters cognitive equity and, consequently, educational equity. This study clearly demonstrates that physical activity fulfills such a role, making the promotion of physical activity among adolescents, particularly those with disadvantaged cognitive ability, not only beneficial for their physical health but also crucially important for enhancing their cognitive ability.

### 4.2 The mediating role of self-education expectations

This study shows that self-education expectations play an independent mediating role between physical activity and adolescent cognitive development. This result is consistent with previous studies, which indicate that adolescents who regularly engage in physical activity tend to have higher educational expectations (Kim and Park, 2015), and higher educational expectations are closely related to enhanced cognitive ability (Liu et al., 2022). From a psychological perspective, self-efficacy theory (Bandura, 1977) provides a theoretical foundation for this phenomenon. The theory suggests that physical activity can enhance adolescents’ sense of self-efficacy, or confidence in their own abilities (Fu et al., 2023). This increased sense of self-efficacy helps lower-achieving adolescents develop a positive self-perception, which extends to their academic expectations, thereby raising their educational expectations. The heightened educational expectations, through pathways such as motivation activation (Brandstätter and Bernecker, 2022) and attention regulation (Anderson, 2013), not only increase adolescents’ likelihood of participating in cognitive activities but also enhance their efficiency in these activities, ultimately promoting the development of their cognitive ability.

### 4.3 The mediating role of learning behaviors

This study found that learning behaviors also play an independent mediating role between physical activity and cognitive ability. Previous studies have shown that physical activity helps improve learning behaviors (Harvey et al., 2018). However, in real life, students, parents, and society often worry that physical activity will take up study time (Ferreira Silva et al., 2022). The results of this study suggest that this concern may be a stereotype. The notion that physical activity reduces study time may be a subjective perception of students and parents. In reality, physical activity often takes up time outside of study hours. For instance, studies have shown that moderate physical activity significantly reduces the time students spend on online games (Li and Jiang, 2023). Therefore, proper scheduling of physical activity not only avoids reducing effective study time but can also improve learning behaviors by reducing time wasted on non-essential activities, thereby increasing the frequency and quality of cognitive activities.

Physiological psychology suggests that changes in brain function are inseparable from physiological changes. Physical activity promotes the secretion of brain-derived neurotrophic factor (BDNF), enhancing the plasticity of brain regions related to learning and memory, such as the hippocampus (Phillips, 2017). This physiological change provides a neural basis for more efficient learning behaviors, improving adolescents’ attention control, information processing, and memory during the learning process. During adolescence, cognitive ability is highly plastic, and learning behaviors are one of the key ways to promote cognitive development (Kolinsky, 2015). Therefore, physical activity not only directly strengthens the neural basis of adolescents’ learning behaviors through physiological mechanisms but also indirectly enhances the “quality” and “quantity” of learning through psychological pathways, such as increasing self-education expectations. As learning behaviors continue to improve, adolescents’ cognitive ability is also significantly enhanced.

### 4.4 Chain mediation of self-educational expectations and learning behaviors

This study shows that physical activity can influence cognitive ability through a chain mediation of self-education expectations and learning behaviors. Previous studies have shown that physical activity helps adolescents build confidence and a positive self-image (Aceves et al., 2020), and this positive sense of self-worth and self-image can enhance their educational expectations. Self-education expectations, as a key psychological factor, not only influence individuals’ goal-setting but also have a profound impact on their behavioral choices. High levels of self-education expectations can continuously motivate individuals in the learning process, strengthening their learning behaviors and thereby enhancing cognitive ability (Hattie et al., 2020). Additionally, studies have found that a lack of physical activity is closely related to cognitive decline (Aichberger et al., 2010), and a reduction in learning behaviors further exacerbates this effect (Zacharopoulos et al., 2021). These findings clearly demonstrate the important roles that self-education expectations and learning behaviors play between physical activity and cognitive ability.

While many studies have focused on fitness factors as key mediators of the effect of physical activity on cognitive ability (Ruiz-Hermosa et al., 2020; Hernández-Jaña et al., 2021), such as the positive effects of aerobic and strength training on fluid intelligence, particularly by promoting the secretion of brain-derived neurotrophic factor (BDNF), enhancing angiogenesis, and synaptogenesis, thereby improving the plasticity of brain regions related to cognitive function (Augusto-Oliveira et al., 2023), this study mainly focuses on the development of crystallized intelligence, a cognitive domain that relies more on the accumulation of learning experiences. Therefore, learning behaviors play a crucial role in the influence of physical activity on cognitive ability. The improvement of crystallized intelligence depends on a continuous learning process, and focusing solely on the direct effects of physical activity while ignoring the role of learning behaviors makes it difficult to establish a clear causal relationship between physical activity and crystallized intelligence.

In conclusion, the main contribution of this study is that it is the first to reveal self-education expectations and learning behaviors as important mediating factors in the promotion of cognitive ability (particularly crystallized intelligence) by physical activity, providing a new theoretical perspective on how physical activity influences adolescent cognitive development through psychological and behavioral pathways.

### 4.5 Implications, limitations and future research

This study confirmed the positive effects of physical activity on adolescents’ cognitive ability and revealed the mediating roles of self-education expectations and learning behaviors, providing valuable insights for both theoretical and practical research on improving adolescents’ cognitive ability. However, this study has certain limitations. First, although it is based on a large and representative sample from a questionnaire survey, selection bias may still exist compared to randomized controlled trials, and the use of propensity score matching (PSM) does not fully eliminate this issue. Future research should adopt more rigorous experimental controls to better address sample selection bias. Second, the questionnaire did not specifically distinguish between the time spent on different types of physical activity, making it difficult to compare the specific effects of various types of physical activity on cognitive ability, which represents a limitation of this study. Therefore, future research should further explore the differential effects of exercise intensity, total load, and different types of physical activity on cognitive ability, in order to more comprehensively examine the mediating roles of self-education expectations and learning behaviors between physical activity and cognitive ability.

## 5 Conclusion

This study shows that physical activity can significantly promote the development of cognitive ability in adolescents. However, the effects of physical activity vary across different groups, with cognitive improvement being more pronounced among adolescents with weaker cognitive ability. In addition to its direct effects, physical activity can also indirectly influence adolescents’ cognitive ability through three mediating pathways: self-education expectations, learning behaviors, and the chain mediation of self-education expectations and learning behaviors. This study, based on a nationally representative sample of Chinese adolescents, explores the role of physical activity in promoting cognitive ability development. The results not only provide strong theoretical support for the formulation of policies aimed at enhancing the cognitive ability of Chinese adolescents but also offer valuable insights for youth sports education practices across various cultural contexts globally. Future research should delve deeper into the specific mechanisms through which different types of physical activities influence cognitive development, to more precisely validate the effects of physical activity and further clarify the mediating roles of self-education expectations and learning behaviors in this process. Additionally, it is worth further investigating how to optimize the effectiveness of physical activity in promoting cognitive development, and its influencing factors, across different social and cultural contexts.

## Data Availability

Publicly available datasets were analyzed in this study. This data can be found here: http://www.isss.pku.edu.cn/cfps/.

## Footnotes

^1^www.isss.pku.edu.cn/cfps/
